# Adrenocortical tumors in children: Sri Lankan experience from a single center, and a mini review

**DOI:** 10.1186/s13256-023-03890-5

**Published:** 2023-04-13

**Authors:** Naotunna Palliya Guruge Chamidri Randika Naotunna, Harankaha Vidanalage Dinendra Nishadini Siriwardana, Balagamage Chamila Lakmini, Dilhara Senani Gamage, Sandani Gunarathna, Malik Samarasinghe, Sanjeewa Gunasekara, Navoda Atapattu

**Affiliations:** 1 Endocrine and Diabetic Unit, Lady Ridgeway Hospital for Children, Colombo, Sri Lanka; 2 Pathology Unit, Lady Ridgeway Hospital for Children, Colombo, Sri Lanka; 3 University Surgical Unit, Lady Ridgeway Hospital for Children, Colombo, Sri Lanka; 4 Department of Surgery, Faculty of Medicine, University of Colombo, Colombo, Sri Lanka; 5 Cancer Institute, Maharagama, Sri Lanka

**Keywords:** Adrenocortical adenoma, Adrenocortical carcinoma, Paediatric cancer

## Abstract

**Background:**

Pediatric adrenocortical tumors include both benign adenomas and highly virulent malignant tumors. However, they are very rare among children. The aim of this study is to evaluate the clinicopathological data of children presenting with adrenocortical tumors and assess their survival in a South Asian population.

**Case presentation:**

This is a retrospective cohort study that includes patients diagnosed with adrenocortical tumors from August 2020 to August 2022 followed-up at Lady Ridgeway Hospital. Seven children were diagnosed with adrenal cortical tumors. Their ages ranged from 10 months to 6.5 years. Five of them were boys. All displayed signs of peripheral precocious puberty. One boy phenotypically had features of Beckwith–Wiedemann syndrome. The median time for diagnosis after the onset of symptoms was 4.4 months. The preoperative diagnosis was based on clinical manifestations, elevated dehydroepiandrosterone sulfate levels, and suprarenal masses on computed tomography. All five boys had right-sided suprarenal masses, while the two girls had them on the left side. All underwent surgery for tumor resection. The diagnosis was confirmed based on the histopathology of the adrenal specimens. Four children had a Wieneke score of 4 or more, suggesting the possibility of adrenocortical carcinoma; however, only two of them behaved as malignant tumors. To date, two children have developed local recurrences within a very short period.

**Conclusion:**

Adrenocortical tumors are uncommon in children, and treatment options are limited. To identify early recurrences, routine clinical, radiological, and biochemical examinations at least once every 6–8 weeks is important.

## Background

Adrenocortical tumors (ACTs) are rare in children, with a reported incidence of 0.2–0.3 cases per million children per year [1]. The highest incidence of ACTs are reported among children younger than 5 years old [2]. ACTs include adrenocortical adenomas (ACA), which are benign, and adrenocortical carcinomas (ACC), which are life threatening [1].

The adrenal gland consists of an outer cortex of glandular tissue and an inner medulla of nervous tissue. The cortex consists of three zones: zona glomerulosa, zona fasciculata, and zona reticularis, with each zone secreting different hormones. Mineralocorticoids (aldosterone), glucocorticoids (mainly cortisol), and androgens are secreted, respectively, from the above zones. Therefore, clinical manifestations differ according to the site of the tumor [2].

Although nonfunctional ACTs are common in adults, functional tumors with hormone hypersecretion are more common in children [3]. The most common presenting symptom is virilization, manifested by pubic hair, enlargement of the penis or clitoris, hirsutism, acne, deepening of the voice, and accelerated growth and skeletal maturation [4, 5]. Cushing syndrome without virilization, feminization, or gynecomastia from excess estrogen, obesity, slowed bone growth from excess steroids, and hyperaldosteronism alone are uncommon manifestations [2, 5]. Nonfunctional tumors may be seen in older children and are often identified incidentally [4].

The definite etiology and pathogenesis of these tumors are not clearly understood [2, 4]. The majority of ACCs are sporadic [6]. Mutations in the p53 gene with familial cancer syndromes and genetic syndromes such as Beckwith–Wiedemann syndrome are a few known causes that can present with ACTs in children [4, 7]. Recent evidence suggests that Insulin-like growth factors (IGF) Binding Protein-2 (IGFBP-2) plays an important role in promoting tumor growth [4].

The pathological differentiation of adenoma from carcinoma has been a challenge. Due to the rarity of the tumor, it has been difficult to establish prognostic factors [7]. Although the Weiss criteria, modified Weiss criteria, and Wieneke index are now available for pathological classification [6], it is still challenging to differentiate ACA from ACC.

Surgery remains the first-line treatment, but in ACC there is a high incidence of recurrence even after complete resection [6]. Adjuvant chemotherapy and radiotherapy have been utilized in managing ACC, yet the 5-year survival rate has been poor [6].

Retrospective data were extracted from the patient database of the Pediatric Endocrinology Unit of Lady Ridgeway Hospital, Colombo, Sri Lanka, from August 2020 to August 2022. The demographic details and clinical data, along with biochemical and imaging findings, were extracted from the clinic records.

## Cases presentation

### Demographics

Seven patients were included in the study (Table 1). Five of them were boys. The median age at diagnosis was 3 years 2 months (ranging from 10 months to 6 years 4 months). Five of the seven (71.4%) were younger than 5 years of age. One boy and one girl were both over the age of 5 years. Five were Sinhalese, the major ethnic group in the country. One was of Sri Lankan Tamil ethnicity, and one was of Moor ethnicity. There was no history of cancer among their family members. All of them were born to nonconsanguineous, healthy parents.

**Table 1:** Demographics and clinical manifestations of patients with adrenocortical tumors

	Frequency (*n*)	Percent (%)
Gender		
Male	5	71.4
Female	2	28.6
Age		
< 1 year	1	14.3
1–5 years	4	57.1
> 5 years	2	28.6
Ethnicity		
Sinhalese	5	71.4
Sri Lankan Tamil	1	14.3
Moor	1	14.3
Clinical manifestations		
Isolated precocious puberty	3	42.9
Isolated Cushing syndrome	0	0
Mixed type	4	57.1
No endocrine abnormality	0	0

All seven patients had features of hormone hypersecretion. They were all virilized at the time of presentation. Four of seven (57.1%) had hypertension and a cushingoid appearance. Clinical manifestations are presented in Table 2. The median interval between the first symptom and the diagnosis was 4.4 months.

**Table 2:** Signs and symptoms of seven children with adrenocortical tumors

Signs and symptoms	Frequency (*n*)	Percent (%)
Pubic hair	7	100
Penile enlargement	5	100
Clitoral enlargement	2	100
Acne	4	57.1
Cushinoid appearance	4	57.1
Hypertension	4	57.1
Breast development	1	50
Height gain	3	42.9
Weight gain	3	42.9
Hypertrichosis	3	42.9
Deepening of voice	1	14.3
Abdominal pain	1	14.3

The oldest boy, who was 6 years 4 months old, had a birth weight of 4.9 kg with asymptomatic hypoglycemia and body asymmetry, which led to a clinical diagnosis of Beckwith–Wiedemann syndrome (BWS). All the other children had an uneventful past medical history.

Both girls, who were 2 years 3 months and 6.5 years old, presented with clitoromegaly. The former had it for 8 months, along with acne and pubic hair. The latter was referred for clitoromegaly of 1 month’s duration, with a recent increment in height, the appearance of pubic hair, and mild breast prominence.

All five boys presented with pubic hair and penile enlargement. Children who were less than 5 years old had stage 2 pubic hair, while the older boy had stage 4 pubic hair.

Four of the seven children had a clinically diagnosed cushingoid appearance with moon-like faces, and all of them had hypertension requiring antihypertensives preoperatively. Three of them required two antihypertensives each to achieve blood pressure control. The mean duration of the antihypertensive requirement postoperatively was 3.5 months.

### Biochemical and imaging findings

All seven patients had elevated dehydroepiandrosterone sulfate (DHEAS) levels, and remarkably, three of the patients had more than the upper limit of the maximum amount that could be analyzed (Table 3). As expected, the testosterone levels were raised in all of them, and the highest level, which was more than threefold the upper limit of normal, was seen in the boy who had voice changes.

**Table 3:** Biochemical and imaging findings

	Patient 1	Patient 2	Patient 3	Patient 4	Patient 5	Patient 6	Patient 7
Age (months)	18	10	76	78	24	35	27
Sex	Male	Male	Male	Female	Male	Male	Female
Preoperative							
DHEAS (µmol/L) (0.9–5.8)	> 40.71	33.42	25.64	21.32	8.66	> 40.71	> 40.71
Testosterone (nmol/L) (< 0.35)	15.5	6.82	5.75	8.72	51.9	10.43	2.78
8 am cortisol (nmol/L)	219	150	412	194	161	341	475
LH/FSH (mIU/L)	< 0.1/0.43	0.1/0.5	< 0.5/0.08	0.2/0.04	< 0.2/0.66	0.1/0.06	< 0.1/
Imaging—CECT							
Size (cm) (AP × Tr × CC)	5.1 × 4.8 × 4.9	3.2 × 3.8 × 2.1	3.4 × 5.3 × 4.2	2.2 × 2.3 × 1.8	2.7 × 1.7 × 2.2	4.9 × 5.4 × 1.7	10 × 6.0 × 5.0
Metastasis at presentation	No	No	No	No	No	No	No
Postoperative							
DHEAS (µmol/L) (0.9–5.8)	< 0.08	0.08	< 0.08	1.47	0.08	0.08	0.08
Testosterone (nmol/L) (< 0.35)	0.5	0.48	0.64	0.82	1.1	0.3	0.34

*DHEAS* dehydroepiandrosterone sulfate; *LH* Luteinizing hormone; *FSH* follicle-stimulating hormone; *CECT* contrast-enhanced computed tomography; *AP* anteroposterior; *TR* transverse; *CC* craniocaudal

LH and FSH levels were in the prepubertal range in all seven patients.

X-ray bone age was advanced in six patients, and only one boy had a delayed bone age of 8 months at the chronological age of 1.5 years. The mean advancement of the bone age was 2 years 6 months (range: 1 year 4 months to 3 years).

An ultrasound scan (USS) of the abdomen detected the tumor, which was later confirmed by contrast-enhanced computed tomography (CECT). All five boys had right-sided adrenocortical tumors, while the two girls had their tumors on the left side. None of the CECT scans identified local or distant metastases at the time of diagnosis. The mean tumor size was 4.5 × 4.1 × 3.12 cm (AP × Tr × CC).

### Treatment

All seven patients underwent tumor resection following the confirmation of the diagnosis by imaging.

### Histopathology

All the resected tumor specimens were histologically analyzed. The Wieneke scoring system was utilized to grade the tumor. Two children had a score up to 2, suggesting a benign evolution, one had a score of 3, while the other four children had a score of 4 or more (Table 4), suggesting the possibility of ACC. All seven patients had atypical mitotic figures.

**Table 4:** Histology: Wieneke score

Histological criteria	Patient 1	Patient 2	Patient 3	Patient 4	Patient 5	Patient 6	Patient 7
Tumor weight > 400 g	No	No	No	No	No	No	No
Tumor size > 10.5 cm	No	Yes	No	No	No	Yes	Yes
Extension in to periadrenal soft tissue or adjacent organs	No	No	No	No	Yes	No	No
Vena cava invasion	No	No	No	No	No	No	No
Venous invasion	No	No	Yes	No	No	Yes	No
Capsular invasion	No	No	Yes	No	Yes	Yes	Yes
Tumor necrosis present 50–60%	Yes	No	Yes	Yes	Yes	Yes	Yes
Mitosis > 15/20 hpf	No	No	Yes	Yes	No	No	Yes
Atypical mitotic figures	Yes	Yes	No	Yes	Yes	No	Yes
Total	2	2	4	3	4	4	5

### Prognosis and follow-up

The mean duration of follow-up for these children is 12.8 months (ranging from 1 month to 24 months). Children who had a Wieneke score of 4 or more were referred to the pediatric oncology team. They all had USS abdomen tests with DHEAS levels three times a month. To date, five of them have had normal studies without evidence of recurrences. None of them had adjuvant therapies. However, two of the patients were identified with local recurrences and distant metastases. One boy (patient 6) presented with cushingoid features 4 months following surgery, and a repeat USS showed tumor recurrence with liver metastasis, which was confirmed by abdomen CECT. He was referred to the oncology unit, and due to extensive metastases, it was decided to give him palliative care. The youngest girl (patient 7) had an uneventful recovery except for having persistently high blood pressure, requiring two antihypertensives, which were tapered off 4 weeks after surgery. Her immediate postoperative DHEAS levels came down to 0.47 mmol/L from > 40 mmol/L, and her USS was found to be normal. She went to the endocrine clinic 6 weeks later, complaining of irritability. Repeat USS revealed local recurrences as well as distant metastases to the liver and spleen. She is currently receiving mitotane-based chemotherapy at the pediatric oncology unit. During the recurrence, both of them had DHEAS levels of more than 40 mmol/L, which previously normalized following initial surgery.

During the surgery, all of them were covered with intravenous hydrocortisone, followed by oral hydrocortisone until full recovery. Hydrocortisone was then tapered off, and after 2 weeks off steroids, the short synacthen test was performed. Six of the seven patients had normal cortisol levels, but the child who had BWS had a poor cortisol response, and he is now on hydrocortisone replacement doses.

The oldest girl showed a height increment 10 months after surgery. Her X-ray bone age was 11 years at 7 years 5 months Biochemistry and imaging confirmed that there was no tumor recurrence. Therefore, further investigations were carried out for precocious puberty, and alarmingly high levels of follicle stimulating hormone (FSH) (96 IU/L) and luteinizing hormone (LH) (33.59 IU/L) were found, pointing toward premature ovarian failure. A karyotype of 46XX excluded Turner syndrome.

## Discussion

Few studies have been published to date, as adrenal tumors are rare in children [1, 5, 8–10]. The largest study findings are from the International Pediatric Adrenocortical Tumor Registry, which involved 254 children for a period of 11 years [5]. However, 79.5% of them originated from southern Brazil, where the ACC incidence is 10–15 times higher than in the rest of the world due to the high prevalence of the P53 mutation [11]. Another study in France retrospectively analyzed 54 patients between 1973 and 1993 [10], while Turkey reported 30 patients from 1970 to 1999 [9]. A recent study published reported on 13 patients diagnosed with ACT over a 10-year period [1]. Our study reports seven patients diagnosed with ACT within a period of 2 years, indicating a higher prevalence compared with the other studies [1, 9, 10].

Compared with all the other studies, our study demonstrates a male predominance [1, 5, 8, 9]. Out of our seven children, we had five boys. The median age of diagnosis in our study was comparable with most of the studies, which were between 3 and 4 years old [5, 8–10], except for one study, which had a median age of 17 months [1]. A bimodal age distribution was observed in studies, where the highest incidence was in children less than 2 years and then peripubertal [5, 8, 10]. However, we did not have a single child who was older than 7 years.

Sutter and others reported that 80–90% of children with ACT presented with endocrine manifestations, and 94% had hormone hypersecretion at the time of diagnosis [4]. Virilization was the most common presentation, alone or in combination with other adrenal hormone excesses. Fifty-seven percent of our study population presented with mixed manifestations (Cushing and virilization), comparable to a recent study from Italy where the majority presented with mixed manifestations (46%), and none had isolated Cushing syndrome [1]. However, 23% of the Italian cohort was asymptomatic at presentation, which is different from our cohort, where all of them had one or more symptoms. Virilization alone (55%) was the first presentation in the majority of the participants in the IPACTR study [5], and 5.5% had Cushing syndrome alone. Nonfunctional tumors were more common in older children [5]. Other less common symptoms included abdominal lumps or pain, fever, volvulus, gynecomastia, feminization, and increased aldosterone secretion alone or in combination with virilization [4, 5, 10].

In published studies, the median time from first presentation to diagnosis was 5–8 months, which is comparable to the 4.4 months in our study.

The majority of children have only local disease at presentation, but some may have regional invasion (10%), while another 15% will have distant metastasis [4]. All our patients had only local disease at the time of presentation.

It is well known that surgical removal of the tumor is the most successful treatment option for ACC [9, 10]. In one study, all children who had ACA only had the local disease and achieved a complete cure after surgical resection [9].

The histological classification of pediatric ACTs is still a challenge. The differentiation between benign and malignant tumors remains controversial. The factors that determine aggressive behavior include older age, a higher mitotic rate, a higher percentage of necrosis, and a large tumor size [12]. Histologically, the Wieneke criteria has been utilized for differentiating between the two, and has been reported to be useful [8, 13, 14]. In our study, those with a Wieneke score of 1–3 indicated benign or indeterminate disease, and clinically behaved in a benign manner, favoring the possibility of an adrenal adenoma. Of the four children who had a Wieneke score of more than 4, which was more in favor of ACC, two behaved clinically as malignant, while the other two children have had a clinically benign course to date. In our cohort, a tumor size greater than 10.5 cm was the most predictive criterion for a malignant course. In our study, the Wieneke score was useful to a certain extent in predicting the clinical outcome. Ki-67 is widely used to predict the outcome of adult adrenal tumors, but it was not used in pediatric scores until recently [15]. A multicenter study was carried out to assess the usefulness of Ki-67, and a scoring system incorporating Ki-67 was implemented in 2019 to guide neoadjuvant treatment for the pediatric ACT, especially following incomplete resection [16]. Adult protocols and experiences shaped treatment guidelines for children [17]. The cornerstone of treatment for stage I–III disease classified using imaging and tumor size and distant spread is surgery, which prolongs survival [5]. Tumor spillage is associated with poor prognosis, and is observed in 20% of initial resections of the tumor and 43% of resections following recurrences, according to previously reported literature [5, 17]. Hence, open adrenalectomy is still the standard of care since laparoscopic resection carries a high risk of rupture and results in more locoregional recurrences [18].

The role of chemotherapy in pediatric ACC is not fully established due to a lack of evidence [17]. Mitotane alone has achieved good results in adults following the complete resection of the adrenal tumor during the last three decades. Mitotane is an insecticide that causes adrenocortical necrosis [19]. Little is known about the use of mitotane in children, even though response rates seem to be comparable to those in adults [5, 20, 21]. The primary challenge in mitotane therapy is related to side effects and the danger of toxicity associated with plasma levels > 20 mg/L, which is thought to be the upper limit of the therapeutic window [17]. The most common side effects include nausea, vomiting, diarrhea, and abdominal pain, followed by less commonly reported cases of lethargy, ataxic gait, depression, and vertigo. Pediatric patients who received mitotane medication for more than 6 months and reached mitotane levels higher than 14 mg/L showed significantly better survival in the nonrandomized single-arm study GPOH-MET 97 [22]. During mitotane therapy, patients may need replacement steroids. Chemotherapy is necessary to manage tumor growth and symptoms of hormone overproduction in patients with severe symptoms or unresectable or recurrent malignancies. An alternative to surgical debulking is cisplatin-based chemotherapy for palliative purposes [17]. ACT has also been treated with other chemotherapeutic drugs, such as 5-fluorouracil, etoposide, cisplatin, carboplatin, cyclophosphamide, doxorubicin, and streptozocin, with varying degrees of success [20, 21]. Radiation therapy in pediatric patients with ACTs has not been well researched because many children with ACTs have germline TP53 mutations, which increase the risk of secondary cancer [17].

The prognosis for pediatric ACC with metastatic disease remains poor despite this multimodal strategy, with an estimated 5-year survival rate below 20% [5, 10, 12, 14, 17].

## Conclusions

Adrenocortical tumors are uncommon in children, with little understanding of clinical classification and treatment options. We present a case series diagnosed over a 2-year period. Five of the seven patients had no recurrence during the period of follow-up.

It is too early to comment on 5-year survival in this cohort, but two of the seven patients already have aggressive metastatic disease. Gonadal failure associated with an adrenal tumor, as was seen in one of our patients, has not been previously reported in the literature.

Regular clinical, radiological, and biochemical evaluation at least every 6–8 weeks is important to detect early recurrences. More sophisticated predictive models for recurrence or malignant risk are useful in determining follow-up or treatment strategies. This could be facilitated with pediatric adrenal tumor registries across the pediatric oncology centers in the world, as the tumor itself is rare and knowledge and experience sharing would improve the outcome of this entity.

## Data Availability

The raw data supporting the conclusions of this article will be made available by the authors upon request.

## References

[1] Miele E, Di Giannatale A, Crocoli A, Cozza R, Serra A, Castellano A (2020). Clinical, genetic, and prognostic features of adrenocortical tumors in children: a 10-year single-center experience. Front Oncol.

[2] Chen QL, Su Z, Li YH, Ma HM, Chen HS, Du ML (2011). Clinical characteristics of adrenocortical tumors in children. J Pediatr Endocrinol Metab [Internet].

[3] Gundgurthi A, Kharb S, Dutta MK, Garg MK, Khare A, Jacob MJ (2012). Childhood adrenocortical carcinoma: case report and review. Indian J Endocrinol Metab..

[4] Sutter JA, Grimberg A (2006). Adrenocortical tumors and hyperplasias in childhood-etiology, genetics, clinical presentation and therapy. Pediatr Endocrinol Rev.

[5] Michalkiewicz E, Sandrini R, Figueiredo B, Miranda ECM, Caran E, Oliveira-Filho AG (2004). Clinical and outcome characteristics of children with adrenocortical tumors: a report from the international pediatric adrenocortical tumor registry. J Clin Oncol.

[6] Gupta N, Rivera M, Novotny P, Rodriguez V, Bancos I, Lteif A (2018). Adrenocortical carcinoma in children: a clinicopathological analysis of 41 patients at the Mayo Clinic from 1950 to 2017. Horm Res Paediatr.

[7] Ribeiro RC, Michalkiewicz EL, Figueiredo BC, Delacerda L, Sandrini F, Pianovsky MD (2000). Adrenocortical tumors in children. Braz J Med Biol Res = Rev Bras Pesqui medicas e Biol..

[8] Wieneke JA, Thompson LDR, Heffess CS (2003). Adrenal cortical neoplasms in the pediatric population: a clinicopathologic and immunophenotypic analysis of 83 patients. Am J Surg Pathol.

[9] Ciftci AO, Şenocak ME, Tanyel FC, Büyükpamukçu N (2001). Adrenocortical tumors in children. J Pediatr Surg.

[10] Teinturier C, Pauchard MS, Brugières L, Landais P, Chaussain JL, Bougnères PF (1999). Clinical and prognostic aspects of adrenocortical neoplasms in childhood. Med Pediatr Oncol.

[11] Lalli E, Figueiredo BC (2015). Pediatric adrenocortical tumors: what they can tell us on adrenal development and comparison with adult adrenal tumors. Front Endocrinol (Lausanne)..

[12] Klein JD, Turner CG, Gray FL, Yu DC, Kozakewich HP, Perez-Atayde AR (2011). Adrenal cortical tumors in children: factors associated with poor outcome. J Pediatr Surg.

[13] Picard C, Orbach D, Dijoud F (2019). Reply to “Pathological prognostication of pediatric adrenocortical tumors: is a gold standard emerging?”. Pediatr Blood Cancer.

[14] Wang Z, Liu G, Sun H, Li K, Dong K, Ma Y (2019). Clinical characteristics and prognosis of adrenocortical tumors in children. Pediatr Surg Int.

[15] Fassnacht M, Dekkers OM, Else T, Baudin E, Berruti A, De Krijger RR (2018). European Society of Endocrinology Clinical Practice Guidelines on the management of adrenocortical carcinoma in adults, in collaboration with the European Network for the Study of Adrenal Tumors. Eur J Endocrinol.

[16] Picard C, Orbach D, Carton M, Brugieres L, Renaudin K, Aubert S (2019). Revisiting the role of the pathological grading in pediatric adrenal cortical tumors: results from a national cohort study with pathological review. Mod Pathol.

[17] Pinto EM, Zambetti GP, Rodriguez-Galindo C (2020). Pediatric adrenocortical tumours. Best Pract Res Clin Endocrinol Metab.

[18] Val P, Ulloa-Aguirre A, Xing Y, Libé R (2015). Adrenocortical carcinoma (ACC): diagnosis, prognosis, and treatment. Front Cell Dev Biol.

[19] Paragliola RM, Torino F, Papi G, Locantore P, Pontecorvi A, Corsello SM (2018). TOUCH MEDICAL MEDIA 62 review endocrine oncology role of mitotane in adrenocortical carcinoma-review and state of the art role of mitotane in adrenocortical carcinoma-review and state of the art. Cit Eur Endocrinol.

[20] Ribeiro RC, Pinto EM, Zambetti GP, Rodriguez-Galindo C (2012). The international pediatric adrenocortical tumor registry initiative: contributions to clinical, biological, and treatment advances in pediatric adrenocortical tumors. Mol Cell Endocrinol.

[21] Rodriguez-Galindo C, Figueiredo BC, Zambetti GP, Ribeiro RC (2005). Biology, clinical characteristics, and management of adrenocortical tumors in children. Pediatr Blood Cancer.

[22] Redlich A, Boxberger N, Strugala D, Frühwald MC, Leuschner I, Kropf S (2012). Systemic treatment of adrenocortical carcinoma in children: Data from the German GPOH-MET 97 trial. Klin Padiatr.

